# Modified Kapandji technique in pediatric displaced distal radius fractures: results in 195 patients

**DOI:** 10.1007/s00590-023-03686-9

**Published:** 2023-08-26

**Authors:** Cristina Bassi, Alexander F. Heimann, Joseph M. Schwab, Moritz Tannast, Ines Raabe

**Affiliations:** Department of Orthopaedic Surgery and Traumatology, HFR Fribourg – Cantonal Hospital, University of Fribourg, Chemin des Pensionnats 2 – 6, 1700 Fribourg, Switzerland

**Keywords:** Kapandji technique, Pediatric, Distal radius fractures

## Abstract

**Purpose:**

The modified Kapandji technique has been proposed for fracture reduction in pediatric displaced distal radius fractures (DDRFs), but evidence is sparse. The purpose of this study was to evaluate our outcomes and complications, critically and systematically, when performing the modified Kapandji technique in pediatric DDRFs. Using this technique since 2011, we asked: (1) What is the quality of fracture reduction using this technique? (2) How stable is fracture alignment with this technique? (3) What are the postoperative complications and complication rates?

**Methods:**

Retrospective observational study of 195 pediatric patients treated with the modified Kapandji technique. Quality of fracture reduction, fixation type (intrafocal, combined, or extrafocal), and coronal/sagittal angulation were recorded at surgery and healing. Perioperative complications were graded. Patients were stratified by fracture (metaphyseal or Salter–Harris) and fixation type, as well as age (≤ 6 years; 6 to 10 years; > 10 years).

**Results:**

Fracture reduction was ‘good’ to ‘anatomical’ in 85% of patients. ‘Anatomical’ fracture reduction was less frequent in metaphyseal fractures (21% vs. 51%; *p* < .001). Mean angulation change was higher in metaphyseal fractures in both the sagittal (*p* = .011) and coronal (*p* = .021) planes. Metaphyseal fractures showed a higher mean change in sagittal angulation during fracture healing for the ‘intrafocal’ group. We observed a 15% overall complication rate with 1% being modified Sink Grade 3.

**Conclusion:**

The modified Kapandji technique for pediatric DDRFs is a safe and effective treatment option. Metaphyseal fractures that do not involve the physis should be treated with extrafocal or combined wire fixation. Complications that require additional surgical treatment are rare.

**Level of evidence:**

Level of evidence IV.

## Introduction

Distal radius fractures are the most common pediatric fractures [1]. Thanks to the exceptional corrective potential of the distal radius, especially in younger children, most distal radius fractures can be treated conservatively. While conservative treatment is well-tolerated, there is the potential risk of secondary fracture displacement, especially when presenting fracture displacement is significant [2, 3] or when considerable risk factors such as obesity are present [4].

The modified Kapandji technique is a method of *K*-wire fracture reduction that has been proposed for pediatric displaced distal radius fractures (DDRF) [5–10]. Evidence of fracture reduction quality, fixation stability, and complications for this technique is sparse, however. In addition, recent biomechanical studies have shown differing levels of stability in distal radius fractures involving the physis versus purely metaphyseal fractures [11].

Our institution has been using the modified Kapandji technique in pediatric DDRFs since 2011. We have used this technique in both physeal and metaphyseal DDRFs when additional assistance is needed to achieve and maintain fracture reduction. The two most common indications are when adequate reduction cannot be achieved in a closed manner, and when closed reduction is adequate but cannot be adequately stabilized in a cast. Given the paucity of the literature on this technique, the purpose of this study was to evaluate our outcomes and complications, critically and systematically, when performing the modified Kapandji technique in pediatric DDRFs. Consequently, we reviewed our series of these cases and asked: (1) What is the quality of fracture reduction using the modified Kapandji technique in pediatric DDRFs operated in our institution? (2) How stable is fracture alignment during healing in these patients? (3) What are the postoperative complications and complication rates?

## Materials and methods

### Study design and settings

This was a retrospective, single-center, observational study (Level of Evidence IV). Institutional review board (IRB) approval was obtained prior to study initiation (IRB Number 2022-00484).

### Patient selection

We reviewed a consecutive series of pediatric patients who presented to our institution between February 2011 and October 2021 with a DDRF and were treated with a modified Kapandji technique fracture reduction and *K*-wire fixation. We included all displaced fractures of the distal radius in children and adolescents up to 16 years of age with radiologic evidence of skeletal immaturity (open growth plates) in the distal radius. Patients were excluded if they underwent successful treatment by closed reduction and cast fixation with no evidence of fracture instability. A fracture was considered unstable if it exhibited any of the following: (1) an initial displacement of more than 50% of the shaft width, (2) sagittal angulation of more than 30° (if patient younger than 10 years) or 10° (if patient older than 10 years), (3) angulation of greater than 10° in the frontal plane, (4) fractures of both bones, or (5) secondary dislocation. Exclusion criteria consisted of previous surgery on the affected extremity, patients treated conservatively, and all other fracture types of the distal forearm.

### Acquisition of study variables

After application of these criteria, 195 study participants were available for further analysis. Due to age-related differences in fracture remodeling potential, study participants were further stratified into three groups: ≤ 6 years (22 patients), 6–10 years (54 patients), > 10 years (119 patients). We also subdivided patients into metaphyseal (140 patients) and Salter–Harris (55 patients) fractures. Our cohort’s demographic information is shown in Table 1.

**Table 1:** Patient demographics

Parameter	Metaphyseal fractures (*N* = 140)	Salter–Harris fractures (*N* = 55)	*p* value
*Age, yr*	10 ± 3	12 ± 3	< .001*
≤ 6 years, *n* (%)	22 (16)	0 (0)	.002*
6 to 10 years, *n* (%)	42 (30)	12 (22)	.082
> 10 years, *n* (%)	76 (54)	43 (78)	.002*
Side, left, *n* (%)	79 (56)	36 (65)	.230
Sex, male, *n* (%)	88 (63)	42 (76)	.064
Height, m	1.43 ± 0.19	1.53 ± 0.18	.009
Weight, kg	37 ± 14	46 ± 15	< .001*
BMI, kg^.^m^−2^	19 ± 3	20 ± 4	.064
*Treatment*			
Intrafocal	67 (48)	39 (71)	.003*
Combined	47 (33)	6 (11)	.002*
Extrafocal	27 (19)	10 (18)	.877
*Additional characteristics*			
Associated ulnar fracture	101 (72)	26 (47)	.001*
Initial Neurovascular impairment	5 (4)	0 (0)	.158
Open fracture	4 (3)	0 (0)	.208

If not otherwise noted, values are mean ± SD

SD, standard deviation; BMI, body mass index

*p < .05

**Fig. 1:**
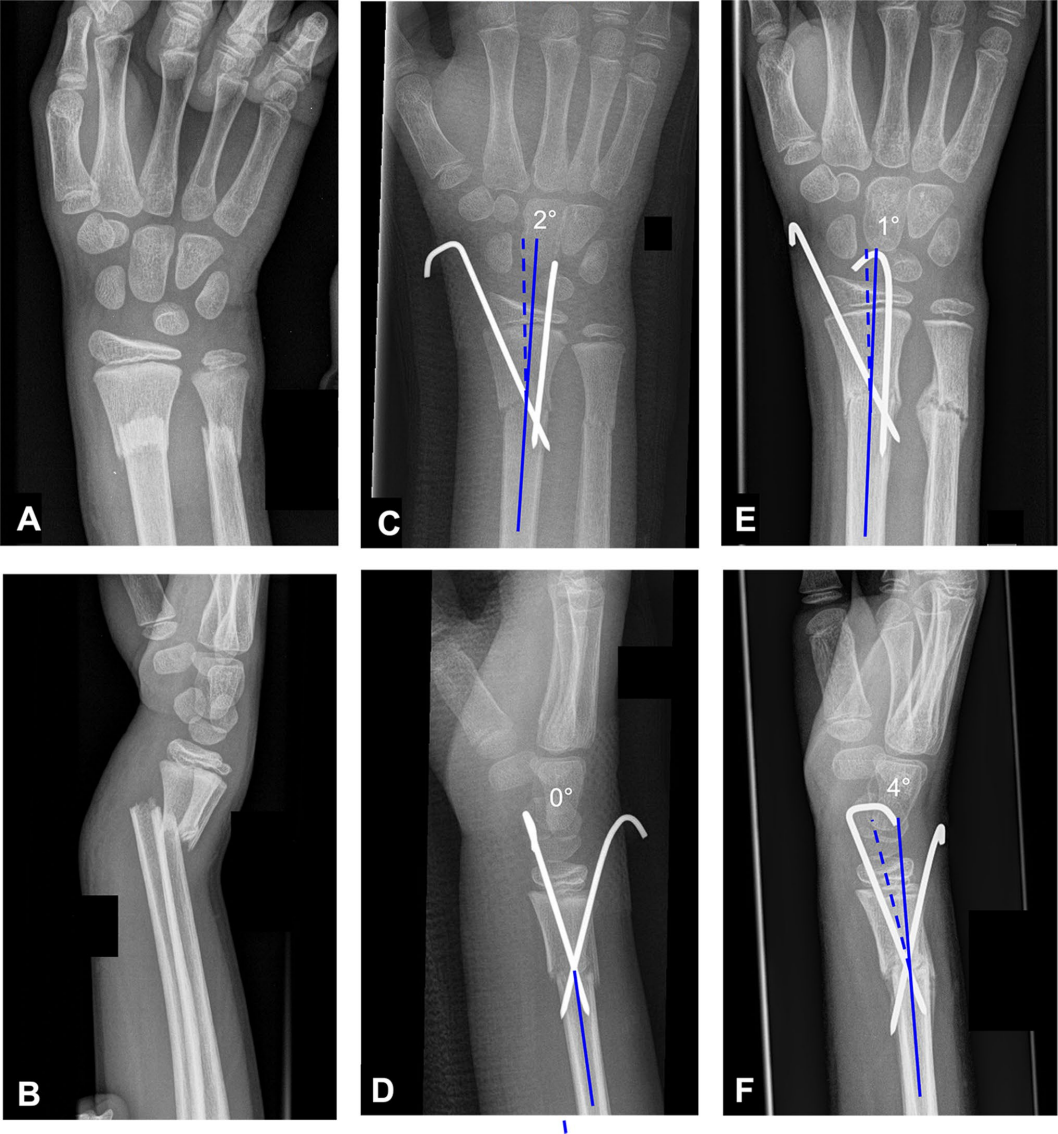
Initial **A** anteroposterior and **B** lateral radiographs of a displaced metaphyseal forearm fracture in a 13-year-old patient. Illustration of the measurement of coronal and sagittal angulation on immediate postoperative radiographs (**C**, **D**) and final healing radiographs (**E**, **F**) after fracture reduction using the modified Kapandji technique and fixation with two extrafocal *K*-wires. In the coronal plane (comparison **C** and **E**) the change in angulation during healing was 1°, while the change in angulation during healing in the sagittal plane (comparison **D** and **F**) was 4°

Fracture classification was made from initial presenting AP and lateral radiographs of the wrist. Fracture reduction was evaluated on postoperative radiographs and rated according to the criteria described by Constantino et al. [12]: no displacement or angulation in both planes was rated ‘anatomical’; < 2 mm of displacement and < 10° of angulation was rated ‘good’; > 2 mm of displacement or > 10° of angulation was rated as ‘fair.’ In addition, both the immediate postoperative radiographs and the final healing radiographs were measured for coronal and sagittal angulation (Fig. 1). Fracture reduction quality was then compared between fracture types.

Operative reports and postoperative clinic notes were examined for evidence of perioperative complications. We abstracted the perioperative complication grading system for orthopedic surgery, as published by Sink et al. [13], for use with pediatric displaced distal radius fractures (see Fig. 4), and we graded our perioperative complications according to this system.

### Surgical technique

**Fig. 2:**
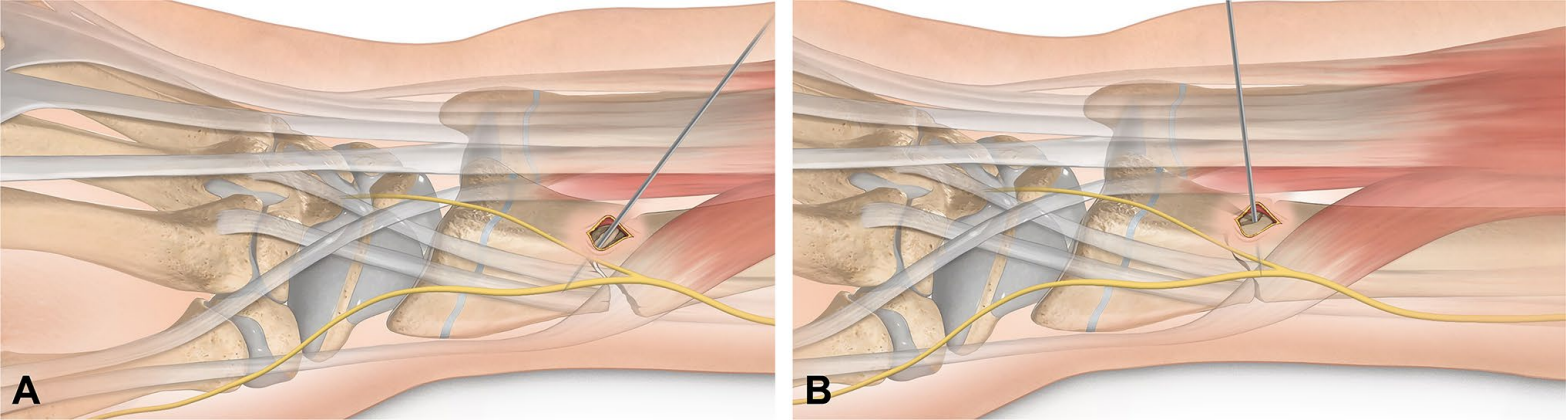
Illustration of the modified Kapandji technique for fracture reduction in displaced distal radius fractures in children. A stab incision is made and **A** a *K*-wire is inserted into the fracture gap. The wire is then used as a lever to **B** reduce the fracture

**Fig. 3:**
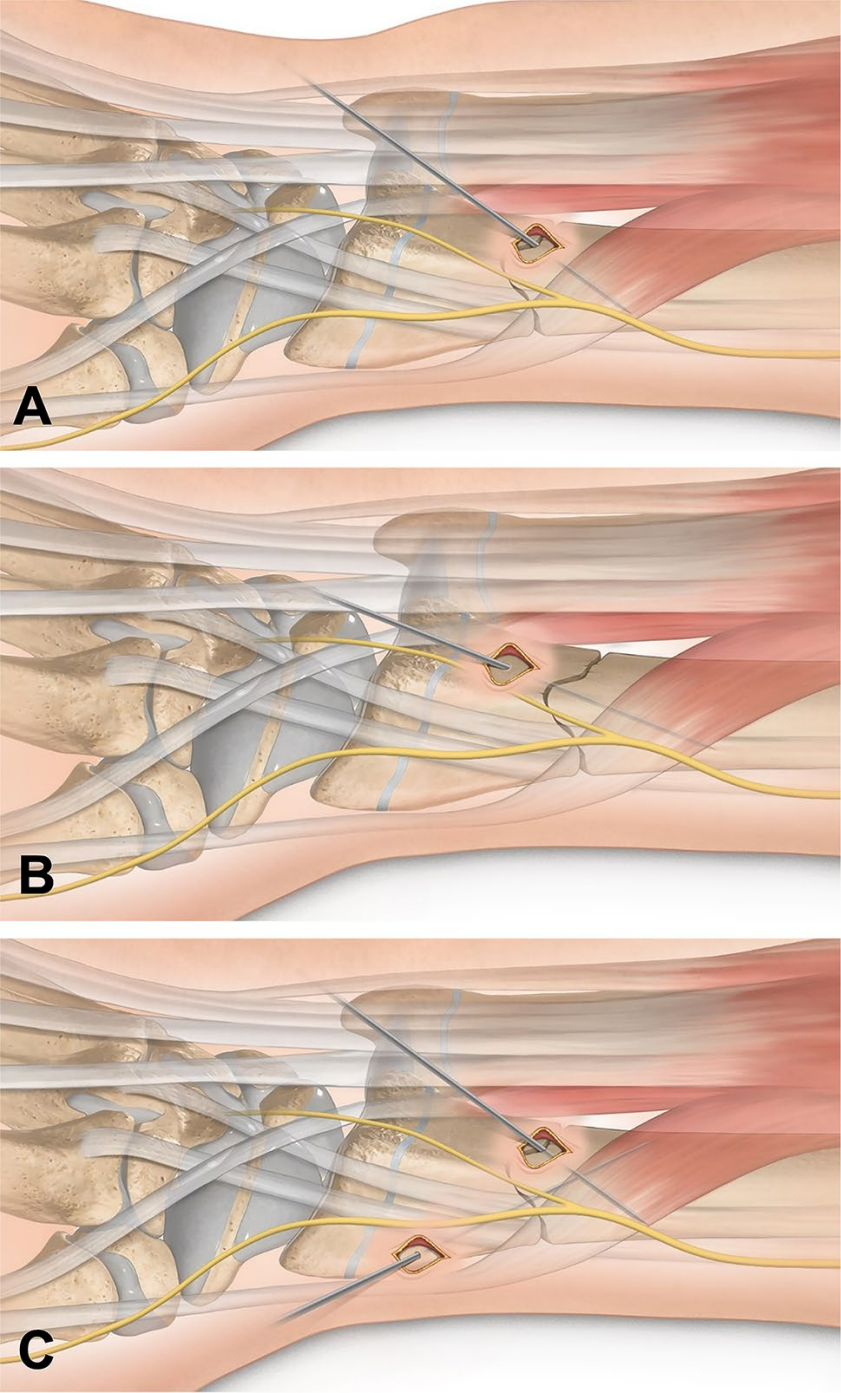
After fracture reduction with the modified Kapandji technique, there are various options for definitive fixation. An intrafocal wire can **A** be driven into the opposite cortex of the proximal fragment. Alternatively, **B** an extrafocal wire can be used, which fixes both the distal and the proximal fragment. Finally, **C** both intrafocal and extrafocal *K*-wires can be used in combination. When fixating the distal fragment, care must be taken to ensure that the *K*-wire enters the bone proximally to the physis, thus sparing it

All operations were performed either by a board-certified orthopedic surgeon (overall 18 different surgeons) or by a surgical resident directly supervised by a board-certified orthopedic surgeon who was scrubbed in the operative procedure. Fracture reduction was performed via stab incisions using the modified Kapandji technique as described by Satish et al. [8] (Fig. 2). Once the fracture was reduced, intraoperative fracture stability testing was performed to determine the need for additional pin fixation. Testing consisted of a wrist flexion–extension maneuver under fluoroscopic imaging. If the fracture redisplaced during this maneuver, a second *K*-wire was inserted either dorsally or radially. This second pin was inserted either intrafocally (i.e., through the visible fracture line and into a stable opposite cortex; Fig. 3A), or extrafocally (i.e., from one stable cortex, across the fracture line, and into the opposite stable cortex; Fig. 3B) in a physeal-sparing manner. Depending on the individual decision of the operator, the intrafocal *K*-wire, originally inserted for fracture reduction, was either left in place and secured into the opposite cortex (see Fig. 3C) or removed if the fracture was stabilized by a second extrafocal pin.

If the fracture pattern included an ulna injury this was also addressed. Unstable, displaced metaphyseal ulna fractures were treated with modified Kapandji technique fracture reduction and subsequent intra- or extrafocal pinning. Ulnar styloid fractures were treated by first assessing the stability of the distal radio-ulnar joint (DRUJ) with the DRUJ dorso-palmar stress test [14]. In the case of an unstable DRUJ, an osteosuture of the ulnar styloid was performed. If the DRUJ was clinically stable, no ulnar fixation was done.

Postoperatively, patients were immobilized in a short arm fiberglass cast for 4 weeks in neutral rotation to mitigate the effects of rotational deforming forces. *K*-wires placed for fracture fixation were bent 90° and left out of the skin. Sterile gauze was placed around the base of the wires as they exited the skin. The cast was windowed around the pin to allow for daily pin cares. Cast immobilization was discontinued and pins were removed in our outpatient clinic at the 4-week postoperative visit.

### Definition of fracture fixation

Fracture fixation was classified as “intrafocal” when all *K*-wires, regardless of absolute number, entered the bone at the fracture site and were fixated only in the stable cortex of the proximal fragment (Fig. 3A). “Extrafocal” was defined as all *K*-wires entering the bone through the stable cortex of one fragment, crossing the fracture, and embedding in the stable opposite cortex of the other fragment. Cases in which both intra- and extrafocal *K*-wires were used for fracture fixation were defined as “combined.”

### Assessment of stability

To assess the stability of the fracture fixation construct we then measured sagittal and coronal angulation through the fracture on both the immediate postoperative radiographs and the first radiographs with evidence of fracture healing. We then grouped these results by our fracture fixation classification and compared any change in sagittal and coronal angulation over time.

### Complications

**Fig. 4:**
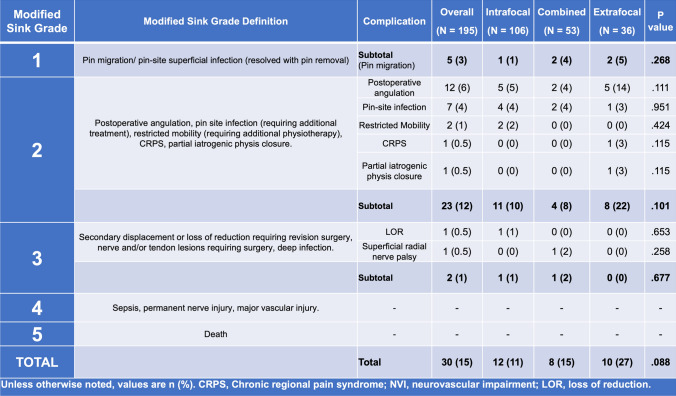
Perioperative complications according to the modified Sink classification

Complications were reviewed and graded according to the classification system presented in Fig. 4. We defined postoperative loss of reduction (LOR) as a change in position of one or more of the following: coronal angulation > 15°, sagittal angulation > 30° (≤ 11 years) or > 20° (> 11 years) [2, 4, 12, 15, 16]. Reduced mobility was defined as a loss of > 10° in wrist flexion/extension and/or pronation/supination compared to the contralateral side at two months postoperatively. Complication rates, as well as the incidence of specific complications, were then compared between fixation groups.

### Statistical analysis

Data were tested for normal distribution using the Kolmogorov–Smirnov test. Depending on distribution, the study data were subsequently analyzed using either a one-way ANOVA or a Kruskal–Wallis test. For binary variables, a chi-square test was performed. A statistical test was considered significant if *p* < 0.05.

## Results

### Quality of fracture reduction using the modified Kapandji technique

Table 2 shows the quality of fracture reduction by fracture type and age subgroup. Overall, fracture reduction was classified as ‘anatomical’ in 30% of patients, ‘good’ in 55%, and ‘fair’ in 15%. ‘Anatomical’ fracture reduction was observed less frequently in metaphyseal fractures compared with Salter–Harris fractures (21% vs. 51%; *p* < 0.001). In addition, ‘fair’ fracture reduction was more common in the metaphyseal fracture group compared with the Salter–Harris fracture group (20% vs. 5%; *p* = 0.013). When comparing age subgroups, the percentage of reductions classified as ‘good’ remained constant with increasing age (range 52% to 55%). Reductions classified as ‘anatomical’ increased from 23 to 33%, while reductions classified as ‘fair’ decreased from 23 to 12% with increasing age.

**Table 2:** Quality of Fracture reduction (according to Constantino et al. [11]) by fracture type and age subgroup

Age subgroup	Overall	Metaphyseal	Salter–Harris	*p* value
*Overall (N* = *195)*				
Anatomical	58 (30)	30 (21)	28 (51)	< .001*
Good	107 (55)	83 (59)	24 (44)	.055
Fair	31 (15)	28 (20)	3 (5)	.013
≤ *6 years (N* = *22)*				
Anatomical	5 (23)	5 (23)	0 (0)	n.a
Good	12 (55)	12 (55)	0 (0)	n.a
Fair	5 (23)	5 (23)	0 (0)	n.a
*6 to 10 years (N* = *54)*				
Anatomical	14 (26)	10 (23)	4 (40)	.265
Good	28 (52)	24 (55)	4 (40)	.410
Fair	12 (22)	10 (23)	2 (20)	.853
> *10 years (N* = *119)*				
Anatomical	39 (33)	17 (22)	22 (51)	.001*
Good	66 (55)	46 (61)	20 (47)	.141
Fair	14 (12)	13 (17)	1 (2)	.017

Unless otherwise noted, values are *N* (%)

*p < .05

### Fracture stability

The mean change in angulation was higher in metaphyseal fractures compared with Salter–Harris fractures in both the sagittal (p = 0.011) and coronal (p = 0.021) planes. When comparing the mean change in sagittal and coronal angulation during fracture healing between the different surgical fixation techniques, no significant differences were found when evaluating either the entire cohort of all fractures, or the subgroup of Salter–Harris fractures. Analysis of metaphyseal fractures, however, showed a higher mean change in sagittal angulation during fracture healing for the ‘intrafocal’ fixation group (Table 3). The presence of a concomitant ulnar fracture did not significantly influence changes in angulation in the sagittal (3 ± 6° vs 2 ± 5°; *p* = 0.302) or coronal (1 ± 3° vs 1 ± 2°; *p* = 0.084) planes.

**Table 3:** Mean change of sagittal and coronal angulation (in °) during fracture healing depending on fracture type and fixation technique

	Total (*N* = 195)	Intrafocal (*N* = 106)	Combination (*N* = 53)	Extrafocal (*N* = 36)	*p* value
*All fractures*					
Mean sagittal change, °	3 ± 6	3 ± 5	2 ± 5	2 ± 7	.169
Mean coronal change, °	1 ± 3	1 ± 3	1 ± 3	2 ± 4	.671

	Total (*N* = 141)	Intrafocal (*N* = 67)	Combination (*N* = 47)	Extrafocal (*N* = 27)	*p* value
*Metaphyseal fractures*					
Mean sagittal change, °	3 ± 6	4 ± 6	2 ± 5	3 ± 8	.042*
Mean coronal change, °	1 ± 3	2 ± 4	1 ± 3	2 ± 4	.282

	Total (*N* = 55)	Intrafocal (*N* = 39)	Combination (*N* = 6)	Extrafocal (*N* = 10)	*p* value
*Salter–Harris fractures*					
Mean sagittal change, °	1 ± 3	1 ± 2	3 ± 6	0 ± 2	.616
Mean coronal change, °	0 ± 1	0 ± 1	1 ± 3	1 ± 1	.083

Unless otherwise noted, values are mean ± SD

SD, standard deviation

*p < .05

### Perioperative complications

There was a total of 30 complications (15%) observed in our series. Five (3%) were Sink Grade 1, 23 (12%) were Sink Grade 2, and 2 (1%) were Sink Grade 3 (Fig. 4). There were no differences noted in the complication rates of the three fixation groups.

## Discussion

Distal radius fractures are the most common pediatric fractures [1]. Surgical treatment may be indicated when fractures exhibit instability with closed reduction. The modified Kapandji technique represents a simple and fast fracture reduction method that can be used to treat DDRFs in children. The reported literature on the modified Kapandji technique in pediatric DDRFs is sparse, especially with respect to the quality of fracture reduction, stability of fixation and associated complications. Our single-center retrospective observational study of a consecutive series of 195 pediatric patients treated with the modified Kapandji technique represents one of the largest series in the literature.

### Reduction quality

In our series, the modified Kapandji technique showed an overall high rate (85%) of good to anatomical fracture reduction. The reduction quality was even higher (95% good to anatomical) in SH fractures. Thanks to the high corrective potential of the pediatric distal radius, injuries with a residual deformity after reduction are likely to resolve without requiring additional treatment, especially in patients under six years of age. Age of the patient, location of the fracture, and amount of deformity are important in considering what is acceptable to treat with observation and what requires revision. Our series demonstrated an overall improvement in the quality of fracture reduction with increasing age. In addition, it is important to note in our series that the modified Kapandji technique not only effectively reduces the fracture, but it also prevents secondary loss of reduction. While conservative treatment reportedly has an incidence of secondary loss of reduction of 20–40%, [2, 17–21] our series demonstrated a loss of reduction in one patient (incidence of 0.5%). This incidence is commensurate with other series that report on the modified Kapandji technique [6, 8], supporting the validity of our findings.

### Stability

All three fixation methods used in our study (intrafocal, combined, and extrafocal) provided adequate fracture stability exhibiting only slight changes in sagittal and coronal alignment during healing in both metaphyseal and Salter–Harris fractures. The observed change of mean sagittal angulation between methods in metaphyseal fractures, although statistically significant, seems clinically minimal. Similar to the reported literature, we observed lower fracture stability in metaphyseal fractures compared to Salter–Harris fractures [11]. It is therefore not surprising that we observed slightly higher mobility of the fixation constructs in the metaphyseal fracture group during follow-up. Based on our findings, we recommend extrafocal pinning, either alone or in combination with intrafocal pinning, when using this technique for pediatric metaphyseal DDRFs. Interestingly, the presence of a concomitant ulnar fracture, when treated according to our protocol, was not associated with an observed difference in postoperative fracture stability in our series.

### Complications

Initially, we were surprised to observe an overall complication rate of 15%, in our series of the modified Kapandji technique. While we were concerned this may represent an unacceptably high complication rate for this type of injury, our overall complication rate is comparable to previously published complication rates after surgical treatment of pediatric DDRFs [22].

Furthermore, our series represents comprehensive reporting and systematic grading of our complications. We adapted the Sink classification system for complications in orthopedic surgery [13] to pediatric DDRFs to provide a comprehensive analysis of our complications and their severity. Twenty-eight of the 30 observed complications were transient complications that fully resolved without significant deviation from the normal postoperative course (i.e., modified Sink Grades 1 and 2). Only 2 of the 30 (7% of all complications, and 1% of all cases) required additional surgical treatment. One case underwent reoperation for loss of reduction and the other to treat a dorsal radial nerve palsy immediately present after surgery. In the latter case the patient had a suture tethering the dorsal radial nerve, and symptoms resolved following suture revision. Patients and their parents should be adequately informed about the risk of pin-associated complications. Overall, the modified Kapandji technique is a safe fracture reduction technique with a low rate of complications (Grade 3 or higher) that require revision surgery.

### Limitations

This study had limitations. First, the patients in the Salter–Harris fracture group were significantly older, heavier, and taller than the patients in the metaphyseal fracture group. This could affect the fracture reduction quality comparison between study groups. However, age at injury appears to be of little importance when comparing postoperative reduction quality. Biomechanical differences in fracture stability between physeal and metaphyseal fractures of the distal radius [11] combined with injury severity are more important. In our study, important indicators for injury severity such as the incidence of initial neurovascular impairment and/or open fractures did not significantly differ between the two groups. Therefore, we believe that our results are still generalizable to these two populations.

Second, our study has the disadvantages associated with a retrospective design. These include an inability to control for confounding factors, multiple treating surgeons, and quality of documentation. While multiple treating surgeons were involved, our institution has a strong continuity of education that ensures significant similarity in approach to these injuries. In addition, we have had a comprehensive electronic health record for over thirty years and are able to systematically document all patient encounters, including complications. The ability to control for confounding factors would be improved by initiating a prospective, randomized, double-blinded controlled trial. Future studies could focus on long-term outcomes of pediatric DDRFs treated by the modified Kapandji technique. Notably, both functional outcomes and patient reported outcome measures (PROMs) would add richness to the overall outcomes data for this procedure.

## Conclusions

In our consecutive case series of pediatric displaced distal radius fractures, the modified Kapandji technique results in high-quality fracture reduction in most patients. Extrafocal pinning, either alone or combined with intrafocal pinning, should be used for unstable metaphyseal fractures. Severe complications requiring additional surgical treatment are rare.
